# Structural and Biochemical Basis for Development of Influenza Virus Inhibitors Targeting the PA Endonuclease

**DOI:** 10.1371/journal.ppat.1002830

**Published:** 2012-08-02

**Authors:** Rebecca M. DuBois, P. Jake Slavish, Brandi M. Baughman, Mi-Kyung Yun, Ju Bao, Richard J. Webby, Thomas R. Webb, Stephen W. White

**Affiliations:** 1 Department of Structural Biology, St. Jude Children's Research Hospital, Memphis, Tennessee, United States of America; 2 Department of Infectious Diseases, St. Jude Children's Research Hospital, Memphis, Tennessee, United States of America; 3 Department of Chemical Biology and Therapeutics, St. Jude Children's Research Hospital, Memphis, Tennessee, United States of America; 4 Integrated Program in Biomedical Sciences, University of Tennessee Health Science Center, Memphis, Tennessee, United States of America; Johns Hopkins University - Bloomberg School of Public Health, United States of America

## Abstract

Emerging influenza viruses are a serious threat to human health because of their pandemic potential. A promising target for the development of novel anti-influenza therapeutics is the PA protein, whose endonuclease activity is essential for viral replication. Translation of viral mRNAs by the host ribosome requires mRNA capping for recognition and binding, and the necessary mRNA caps are cleaved or “snatched” from host pre-mRNAs by the PA endonuclease. The structure-based development of inhibitors that target PA endonuclease is now possible with the recent crystal structure of the PA catalytic domain. In this study, we sought to understand the molecular mechanism of inhibition by several compounds that are known or predicted to block endonuclease-dependent polymerase activity. Using an *in vitro* endonuclease activity assay, we show that these compounds block the enzymatic activity of the isolated PA endonuclease domain. Using X-ray crystallography, we show how these inhibitors coordinate the two-metal endonuclease active site and engage the active site residues. Two structures also reveal an induced-fit mode of inhibitor binding. The structures allow a molecular understanding of the structure-activity relationship of several known influenza inhibitors and the mechanism of drug resistance by a PA mutation. Taken together, our data reveal new strategies for structure-based design and optimization of PA endonuclease inhibitors.

## Author Summary

Seasonal and pandemic influenza have enormous impacts on global public health. The rapid emergence of influenza virus strains that are resistant to current antiviral therapies highlights the urgent need to develop new therapeutic options. A promising target for drug discovery is the influenza virus PA protein, whose endonuclease enzymatic activity is essential for the “cap-snatching” step of viral mRNA transcription that allows transcripts to be processed by the host ribosome. Here, we describe a structure-based analysis of the mechanism of inhibition of the influenza virus PA endonuclease by small molecules. Our X-ray crystallographic studies have resolved the modes of binding of known and predicted inhibitors, and revealed that they directly block the PA endonuclease active site. We also report a number of molecular interactions that contribute to binding affinity and specificity. Our structural results are supported by biochemical analyses of the inhibition of enzymatic activity and computational docking experiments. Overall, our data reveal exciting strategies for the design and optimization of novel influenza virus inhibitors that target the PA protein.

## Introduction

Influenza viruses can cause sporadic global pandemics, and they can result in high mortality rates such as the 1918 pandemic that resulted in 30 to 50 million deaths worldwide [1]. The recent 2009 pandemic was caused by a novel H1N1 virus that originated in swine [2], but of more concern is the impending threat of the highly pathogenic avian influenza H5N1 viruses that cause mortality rates approaching 60% when transmitted to humans [3]. Although H5N1 viruses have yet to naturally acquire the capacity for efficient human-to-human transmission, this has recently been demonstrated in animal models [4], [5] and they remain an ever-present threat due to their continued circulation in avian species. The development of a new vaccine requires several months, and effective antiviral therapies are therefore important at the beginning of a fast-spreading pandemic. Antivirals that target the M2 ion channel (amantadine and rimantadine) or neuraminidase (zanamivir and oseltamivir) have proven to be effective at reducing the severity of illness (reviewed in [6]), but the rapid emergence of resistant strains has highlighted the need for new therapeutic options [7].

Influenza virus contains a negative-strand segmented RNA genome comprising eight ribonucleoprotein assemblies. The RNA-dependent RNA polymerase (RdRp) catalyzes both the transcription and replication steps that are essential in the virus life cycle. The RdRp is a heterotrimeric complex comprising subunits PA, PB1, and PB2 that associates with the 3′ and 5′ ends of each RNA genome segment [8], [9]. Translation of viral mRNAs by the host ribosome requires 5′ capping, and the necessary mRNA caps are cleaved or “snatched” from host pre-mRNAs. This “cap-snatching” mechanism begins with the binding of PB2 to the cap of a host pre-mRNA, followed by the cleavage of the pre-mRNA by the endonuclease functionality [10], [11], [12]. The resulting 10- to 14-residue cap-containing oligonucleotide is then used as a primer for viral mRNA transcription by PB1 [13], [14].

The endonuclease activity is an excellent target for the development of new anti-influenza inhibitors [15], and recent crystallographic studies have facilitated this approach. Two groups found that the endonuclease activity resides not in PB1 as previously suggested [11] but in an independently folded N-terminal domain of PA (PA_N_) [16], [17]. This explains previous findings that PA-specific siRNA can down-regulate viral mRNA production and block virus replication in cell culture [18]. The crystal structures revealed that PA_N_ is a member of the PD-(D/E)XK nuclease superfamily, although there was disagreement as to whether there is a single magnesium (Mg^2+^) ion in the active site [17] or two manganese (Mn^2+^) ions [16]. However, PA_N_ has greater thermal stability and higher endonuclease activity in the presence of Mn^2+^ ions than other divalent cations [16], and isothermal titration calorimetry (ITC) [19] and earlier studies [20] also support the presence of two Mn^2+^ ions.

During the past 5 years, structural studies have revealed that the influenza RdRp comprises multiple, independently-folded, sub-domains with defined functionalities, and the PA_N_ domain structure is particularly important with implications for structure-based drug discovery [10], [16], [17], [21], [22], [23], [24], [25]. Mutational analyses support the idea that the PA_N_ domain is a valuable vehicle for drug discovery [12], [17], [19]. Previous studies have reported inhibitors of influenza transcription and/or endonuclease activity, but there are no structural data demonstrating their molecular mechanisms [15], [26], [27], [28], [29], [30]. Here, we present crystal structures of PA_N_ from strain A/Vietnam/1203/2004 (H5N1) in complex with six known or predicted inhibitors that allow us to precisely describe their interactions with the PA_N_ active site. In an accompanying article by Kowalinski and coworkers, structures of a complementary set of inhibitors in complex with PA_N_ from strain A/California/04/2009 (H1N1) are reported [31]. Together, our structures provide a molecular explanation for the structure-activity relationship (SAR) of several related influenza inhibitors, reveal the mechanism of drug-resistance by a PA mutation, and provide a solid basis for future structure-based drug discovery efforts.

## Results

### Structural Analysis of a Modified PA_N_ Domain

**Figure 1:**
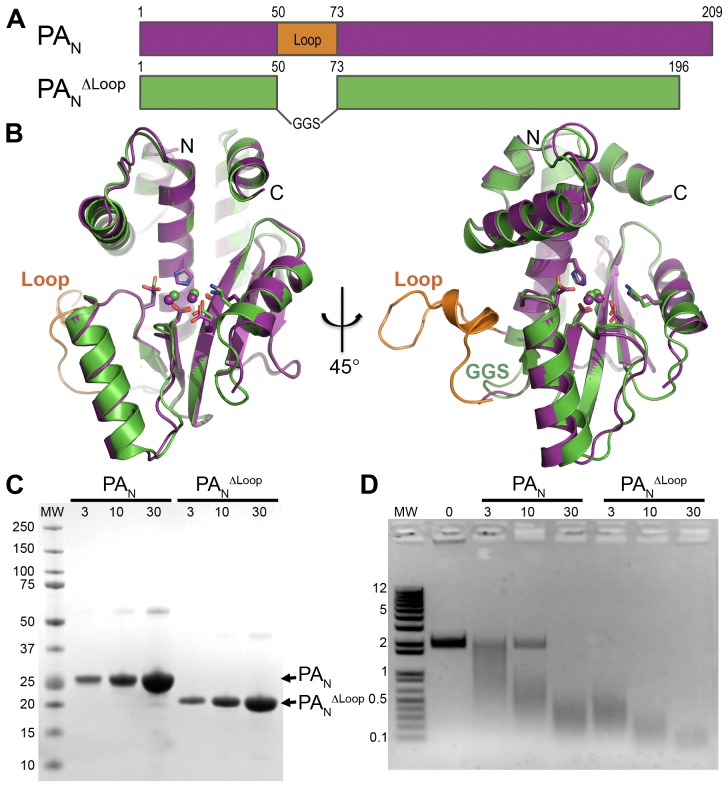
Crystal structure and endonuclease activity of PA_N_^ΔLoop^. (**A**) Schematic of PA_N_ (magenta) and PA_N_^ΔLoop^ (green), and location of the 22-residue loop (orange) replaced by a Gly-Gly-Ser linker in the PA_N_^ΔLoop^ construct. (**B**) Two orthogonal views of the overlay of the crystal structures of PA_N_ and PA_N_^ΔLoop^, colored as in **A**. Key active site residues are shown in stick representation, the paired manganese ions are shown as spheres, and the N- and C-termini are labeled. The coordinates for PA_N_ are from PDB entry 2W69. The atomic coordinates and structure factors for PA_N_^ΔLoop^ have been deposited in the Protein Data Bank as PDB entry 4E5E. (**C**) Coomassie-stained SDS-PAGE of PA_N_ and PA_N_^ΔLoop^ showing the amount of protein (µM) in a 10 µl endonuclease activity assay reaction. Molecular weight (MW) markers (kD) are shown on the left. (**D**) Endonuclease activity assay with PA_N_ and PA_N_^ΔLoop^. Single-stranded DNA plasmid M13mp18 was incubated with increasing concentrations (µM) of PA_N_ or PA_N_^ΔLoop^. Reactions products were resolved on a 1.0% agarose gel stained with ethidium bromide. Molecular weight (MW) ladder (kb) is shown on the left.

The structure of the PA_N_ domain has been reported in two studies [16], [17], but neither construct was considered suitable for drug discovery. In one structure, a 22-residue loop of one PA_N_ molecule packs into the active site of a neighboring molecule [16] making it unavailable for inhibitor binding. In the second structure, although these loop residues are disordered and the PA_N_ active site is suitably exposed, we were unable to reproduce these crystals at high resolution [17]. We therefore designed a new truncated construct of PA_N_, termed PA_N_^ΔLoop^, from strain A/Vietnam/1203/2004 (H5N1) (Fig. 1A), in which the loop is replaced by a Gly-Gly-Ser linker and which ends at residue 196, the last visible residue in both of the crystal structures. PA_N_^ΔLoop^ readily crystallized in a new crystal form that diffracted to 2.05 Å (Table 1, PA_N_^ΔLoop^–Apo) with four molecules in the asymmetric unit and all active sites exposed (Fig. S1A). The PA_N_^ΔLoop^ structure is essentially identical to the previously reported structures of PA_N_ (backbone alpha-carbon RMSD of 0.45 Å). Importantly, the active site residues are virtually superimposable (Fig. 1B), two metal ions are clearly present (Fig. 1B), and the dose-dependent endonuclease activity is unaffected by the truncations (Fig. 1C, 1D). This suggests that the function of the loop is architectural rather than catalytic, presumably to mediate interactions with another subunit of the influenza RdRp or with a host cell factor.

**Table 1:** Crystallographic statistics.

Data collection^a^							
Crystal	PA_N_-Apo	PA_N_-compound 1	PA_N_-compound 2	PA_N_-compound 3	PA_N_-compound 4	PA_N_-compound 5	PA_N_-compound 6
Space group	C222_1_	C222_1_	C222_1_	C222_1_	C222_1_	C222_1_	C222_1_
*a*, *b*, *c* (Å)	126.4, 133.9, 126.4	126.6, 134.3, 126.7	126.9, 133.7, 126.8	126.1, 134.8, 125.9	126.3, 134.8, 126.5	126.1, 134.1, 126.6	126.5, 133.5, 126.4
α, β, γ (°)	90.0, 90.0, 90.0	90.0, 90.0, 90.0	90.0, 90.0, 90.0	90.0, 90.0, 90.0	90.0, 90.0, 90.0	90.0, 90.0, 90.0	90.0, 90.0, 90.0
Resolution (Å)	50.0–2.05 (2.12–2.05)	50.0–2.40 (2.49–2.40)	50.0–2.65 (2.74–2.65)	50.0–2.15 (2.23–2.15)	50.0–2.95 (3.06–2.95)	50.0–2.35 (2.43–2.35)	50.0–2.50 (2.59–2.50)
*R*_merge_	0.063 (0.457)	0.072 (0.488)	0.072 (0.495)	0.065 (0.511)	0.071 (0.488)	0.073 (0.484)	0.081 (0.528)
*I*/*σI*	31.0 (3.0)	35.3 (4.5)	36.9 (5.2)	27.3 (4.3)	28.7 (2.2)	32.4 (5.9)	37.1 (4.7)
Completeness (%)	99.6 (96.3)	99.9 (100.0)	99.9 (99.9)	99.8 (100.0)	96.6 (74.4)	99.8 (100.0)	99.9 (100.0)
Redundancy	8.0 (5.7)	10.3 (9.5)	12.4 (12.0)	9.0 (7.4)	8.4 (5.3)	12.0 (11.3)	10.3 (9.6)
**Refinement**							
Resolution (Å)	50.0–2.05	50.0–2.40	50.0–2.65	50.0–2.15	50.0–2.95	50.0–2.35	50.0–2.50
No. reflections	88,796	56,039	41,126	76,756	29,768	58,174	52,052
*R*_work_/*R*_free_ b	0.226/0.260	0.242/0.288	0.244/0.285	0.251/0.285	0.320/0.368	0.250/0.290	0.239/0.288
Ramachandran (%)							
Favored	98.7	98.3	98.5	98.6	96.9	98.2	98.7
Allowed	1.3	1.7	1.5	1.4	3.1	1.8	1.3
Outliers	0.0	0.0	0.0	0.0	0.0	0.0	0.0
Rms deviations							
Bond lengths (Å)	0.008	0.010	0.008	0.011	0.006	0.010	0.011
Bond angles (Å)	0.992	1.148	1.063	1.156	0.877	1.164	1.169

a Data were collected from a single crystal. Values for the highest-resolution shell are shown in parentheses.

b *R*_free_ was calculated using 5% of the reflections.

### Metal Ion Binding in the PA_N_ Active Site

Previous structural studies raised the question as to whether there is a single Mg^2+^ ion [17] or two Mn^2+^ ions [16] in the PA_N_ active site. Because of this uncertainty, we included both 10 mM MgCl_2_ and 5 mM MnCl_2_ in our crystal soaking solutions. We eventually modeled two Mn^2+^ ions into the active sites of all of our structures for the following reasons. First, PA_N_^ΔLoop^–Apo crystals soaked in a solution containing only 5 mM MnCl_2_ revealed strong electron density in both metal sites (Fig. S1B). Second, refinements of all our structures consistently favored Mn^2+^ over Mg^2+^ ions to account for the observed electron densities. Third, ITC studies have shown that two Mn^2+^ ions bind tighter than one Mg^2+^ ion [19]. Finally, in the accompanying article by Kowalinski and coworkers, a strong anomalous signal for Mn^2+^ was observed in both metal sites when diketo inhibitors or mononucleotides are bound to PA_N_ [31].

### Inhibition of PA_N_ Endonuclease Activity by Three Known Polymerase Inhibitors

**Figure 2:**
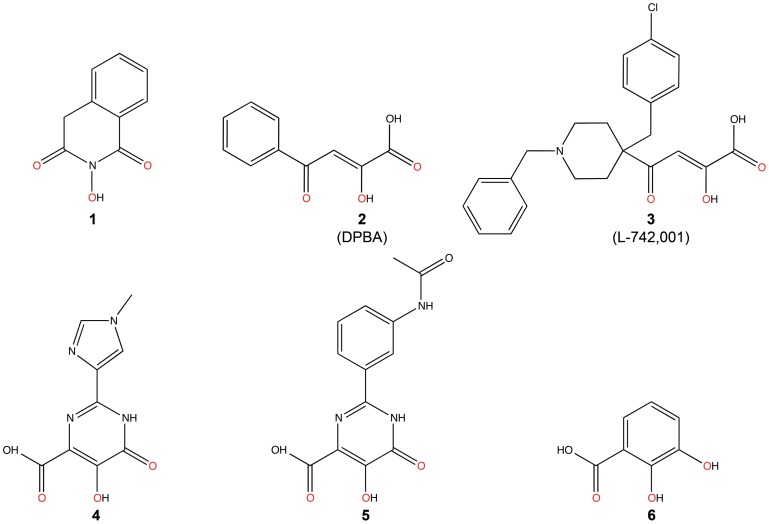
Chemical structures of compounds used in this study. Oxygen atoms that coordinate manganese ions in the active site of PA_N_^ΔLoop^ are colored red.

**Figure 3:**
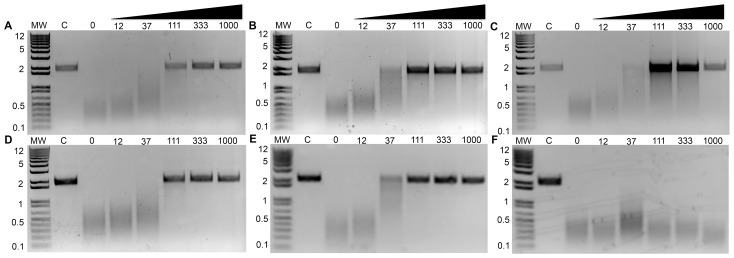
Inhibition of PA_N_ endonuclease activity by known and predicted inhibitors. Compounds **1**–**6** (**A**–**F**, respectively) were incubated at increasing concentrations (µM) with 15 µM PA_N_ and single-stranded DNA plasmid M13mp18. Reaction products were resolved on a 1.0% agarose gel and stained with ethidium bromide. Control lanes (‘C’) contained no PA_N_ in the reaction mixture. Molecular weight (MW) ladder (kb) is shown on the left.

We first investigated three known inhibitors of the influenza RdRp, compounds **1**–**3** (Fig. 2). Compound **1** is an *N*-hydroxyimide that has been shown to inhibit transcription *in vitro* [29], and it is structurally related to Flutimide that was found to specifically inhibit transcription, endonuclease activity, and influenza virus replication [30]. Compounds **2** (2,4-dioxo-4-phenylbutanoic acid, or DPBA) and **3** (L-742,001) are members of a series of 4-substituted 2,4-dioxobutanoic acids that were found to inhibit both transcription and endonuclease activities by purified RdRp *in vitro* [15]. Compound **3** is one of the most potent inhibitors of influenza transcription, and it exhibits dose-dependent inhibition of viral replication in cell culture (IC_50_ value 0.35 µM) and in mice [15], [26]. Purified, recombinant PA_N_ was incubated with single-stranded DNA substrate and increasing concentrations of **1**, **2**, and **3** (Fig. 3), and each inhibited PA_N_ enzymatic activity in a dose-dependent manner. While this activity has been reported for **2** [16], this is the first evidence that **1** and **3** also inhibit the isolated PA_N_ domain.

**Figure 4:**
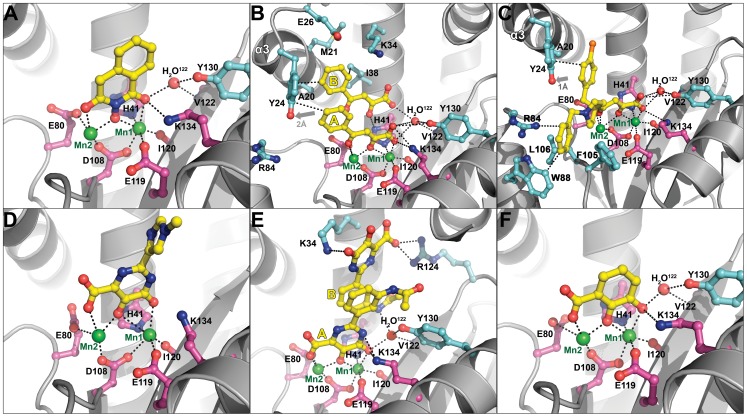
Crystal structures of PA_N_^ΔLoop^ bound to compounds 1–6 (A–F, respectively). PA_N_^ΔLoop^ is shown as gray cartoon. Compounds are shown as ball-and-stick models and are colored yellow (carbon), blue (nitrogen), red (oxygen), and orange (chlorine). Manganese ions (Mn1 and Mn2) are shown as green spheres. The carbon atoms of key active site residues are colored magenta, and the carbon atoms of other residues interacting with the compound or discussed in the text are colored cyan. An ordered water molecule (H_2_O^122^) is shown as a red sphere. Black dotted lines represent molecular interactions less than 3.2 Å away. In the case of compounds **2** and **5**, two molecules (yellow labels A and B) are bound in the PA_N_^ΔLoop^ active site. Gray arrows (panels **B** and **C**) show the movement of helix-α3 residue Tyr24 compared with the structure of PA_N_^ΔLoop^-Apo. The atomic coordinates and structure factors for PA_N_^ΔLoop^ bound to compounds **1**–**6** have been deposited in the Protein Data Bank as PDB entries 4E5F, 4E5G, 4E5H, 4E5I, 4E5J, and 4E5L, respectively.

To investigate the mechanisms of action of **1**, **2**, and **3**, we determined their co-crystal structures with PA_N_^ΔLoop^ (Table 1). Clear difference electron density showed each compound adjacent to the active site Mn^2+^ ions (Figs. 4, S2). In each structure, the three adjacent and planar oxygen atoms on the inhibitor chelate the two Mn^2+^ ions in a pairwise fashion such that the central oxygen atom is shared by the ions. Thus, Mn^2+^ ion 1 (Mn1) is octahedrally coordinated to His41, Asp108, Glu119, Ile120 (carbonyl) and two oxygen atoms in the inhibitor, and Mn2 is tetrahedrally coordinated by Glu80, Asp108, and two oxygen atoms in the inhibitor. The side oxygen atom of the former pair also forms hydrogen bonds to Lys134, a key catalytic residue [12], [16], [17], [19], and an ordered water molecule (H_2_O^122^).

The orientation of compound **1** in the active site was not entirely clear. Two of the four molecules in the asymmetric unit showed convincing electron density for the orientation shown in Figures 4A and S2A, while the orientations of the other two molecules were ambiguous. This ambiguity may reflect the weak electron density, possibly due to the poor solubility of **1** in the crystal soak solution. Alternatively, the benzene ring forms no obvious interactions with PA_N_^ΔLoop^, and **1** may be free to adopt two alternate docking modes.

Compound **2** has also been structurally characterized in complex with the La Crosse virus endonuclease, and it engages the two-metal active site in the same fashion [32]. However, in the PA_N_ complex, two copies of the molecule are bound in the active site. Molecule A engages the Mn^2+^ ions and molecule B π-stacks onto molecule A in a parallel fashion via the phenyl group and the planar side chain (Figs. 4, S2). This arrangement was present in all four active sites in the asymmetric unit. The carboxyl group of molecule A forms a salt bridge to Lys134 and hydrogen bonds to metal-coordinating residues His41, Glu119, and Ile120 (carbonyl) and to H_2_O^122^. Molecule B engages a pocket comprising Ala20, Met21, Glu26, Lys34, and Ile38 (Figs. 4B), and its carboxyl side chain also forms hydrogen bonds to His41 and H_2_O^122^ in a fashion similar to that of molecule A. The phenyl groups of both molecules form an edge-to-face interaction with the side chain of Tyr24 that is pushed out approximately 2.0 Å in comparison with the PA_N_^ΔLoop^-Apo structure. This suggests that the binding of compound **2** involves an induced-fit mechanism (Figs. 4, S3), and the relatively high B-factors in helix-α3 that contains Tyr24 reveal that this region is suitably mobile (Fig. S3).

Kowalinski and coworkers also describe the structure of PA_N_ bound to compound **2** and reveal an identical mode of binding [31]. However, they did not observe the second bound molecule, and we suggest that this is due to the higher concentration of **2** used in our structural studies. To confirm the stoichiometry of binding of compound **2** at the higher concentration, we carried out ITC experiments (Fig. S4). Analysis of the data strongly supports a 1∶2 molar ratio for PA_N_∶compound **2** (N = 1.86, Fig. S4A), and an alternative analysis using a sequential binding model (Fig. S4B) also supports the second bound molecule of **2**, albeit with a nearly 100-fold lower affinity. These ITC analyses are therefore consistent with the structures in both studies where one or two molecules bind PA_N_ depending on the concentration of compound **2**.

Compound **3** binds in a similar orientation as **2**, with the carboxylic acid interacting with Lys134 (Fig. 4C). The increased potency of **3** is likely due to the additional interactions formed by the benzylpiperidine and chlorobenzyl groups that splay in opposite directions perpendicular to the dioxobutanoic acid. The chlorobenzyl group engages the pocket occupied by the phenyl groups of molecules A and B in **2** (Fig. 4). The piperidine moiety directs the benzyl group into a narrow pocket comprising Arg84, Trp88, Phe105, and Leu106 (Fig. 4C). Although the electron density for **3** was relatively poor (Fig. S2), our model is supported by several lines of evidence. First, molecular docking of **3** into the PA_N_ active site yields a strikingly similar orientation to that found in our crystallographic model (Fig. S5). Second, the chlorobenzyl group causes a similar movement in Tyr24 that is seen for **2**, which suggests that **3** also binds via an induced-fit mechanism (Figs. 4, S3). Finally, mutation of Thr20 to alanine within the pocket occupied by the chlorobenzyl group caused a 3-fold reduction in virus inhibition in cell culture and a 2–3-fold reduction in inhibition of transcription by **3** (L-742,001) [18]. In our PA_N_^ΔLoop^ construct, residue 20 is naturally an alanine, and a reduced affinity for **3** could explain the weak electron density for the chlorobenzyl group. We hypothesize that the larger threonine side chain mediates tighter interactions with the chlorobenzyl group and thereby increases affinity and inhibition.

Kowalinski and coworkers report the structure of PA_N_ bound to compounds related to **3** [31], but the most closely-related compound (R05-2) adopts a significantly different orientation. The cyclohexane group of R05-2 is rotated 180° to coincide with the chlorobenzyl group of **3**, and the chlorobenzyl group of R05-2 enters a completely different pocket. The orientation of R05-2 is incompatible with the electron density of **3** and the reverse is also true [31]. The difference in conformations is not entirely surprising because Kowalinski and coworkers demonstrate that a similar compound (R05-3) binds in two distinct conformations [31]. We suggest that these compounds may adopt various conformations within the large PA_N_ active site cleft depending on the microenvironment.

### Prediction and Characterization of Three Additional PA_N_ Endonuclease Inhibitors

Two-metal active sites similar to the one observed in PA_N_ are present in many enzymes that process nucleic acids, and they mediate a common catalytic reaction [33]. Raltegravir is an antiretroviral drug developed to treat HIV infections, and it targets the two-metal active site of HIV integrase [34]. The drug is built around a central pyrimidinol ring scaffold that contains in its plane three adjacent oxygen atoms similar to compounds **1**–**3**, and these oxygen atoms also coordinate the two-metal center in the active site of foamy virus integrase [35], [36]. In keeping with our hypothesis that the pyrimidinol scaffold can serve as a general inhibitor of two-metal enzymes [37], we predicted that compounds **4** and **5**, which also contain the pyrimidinol scaffold (Fig. 2), would inhibit PA_N_ activity, and showed this to be the case (Fig. 3). Structural characterization of the two compounds bound to PA_N_ (Table 1) confirmed their interaction with the two Mn^2+^ ions, but we were surprised to find that their carboxyl groups are not in same location as the carboxyl group in compounds **2** and **3** (Figs. 4, S2). Compared with **2** and **3**, the pyrimidinol scaffold is flipped by 180° and there is no electrostatic interaction between the carboxyl groups and Lys134. We suggest that the flipped orientation of compounds **4** and **5** is necessary to maintain the optimal metal coordination for Mn1 (see discussion).

The imidazole and phenyl moieties of compounds **4** and **5**, respectively, show no obvious interactions with the PA_N_ active site cleft, but similar to what we observed with compound **2**, a second molecule (B) of compound **5** π-stacks onto molecule A (Figs. 4E, S2E). Molecule B is rotated 180° compared to molecule A and they interact via π-stacking interactions between the pyrimidinol and phenyl groups. Molecule B is further stabilized by hydrogen-bonding and ionic interactions with Lys34 and Arg124 (Fig. 4E). Attempts to determine the binding stoichiometry of compound **5** using ITC were not successful due to compound solubility problems, but similar to compound **2**, the electron density is unequivocal.

Finally, two recent studies have identified several compounds, including marchatins, green tea catechins, and dihydroxy phenethylphenylphthalimides, that inhibit PA_N_ endonuclease activity and influenza virus growth [27], [28], [38], [39]. The common moiety in these inhibitors is a dihydroxyphenethyl group, and we predicted that dihydroxybenzoic acid (compound **6**), which contains this moiety and has oxygen atoms in positions similar to those in compounds **4** and **5**, would be able to bind and inhibit PA_N_ (Fig. 2). Although the compound shows little ability to inhibit PA_N_ endonuclease activity (Fig. 3F), we were able to determine the structure of **6** bound to PA_N_ at a resolution of 2.50 Å (Table 1). Compound **6** interacts with the two Mn^2+^ ions in the same orientation as the pyrimidinol scaffold (Figs. 4F, S2F). These data suggest that the dihydroxyphenethyl group binds to the PA_N_ active site in the same manner as **4** and **5**, but that additional interactions available in the marchatins, green tea catechins, and dihydroxy phenethylphenylphthalimides are required to inhibit PA_N_ activity. Indeed, Kowalinski and coworkers report the structure of PA_N_ bound to the green tea catechin EGCG and this reveals these additional interactions [31].

### Structural Basis of the SAR of Known Inhibitors

**Figure 5:**
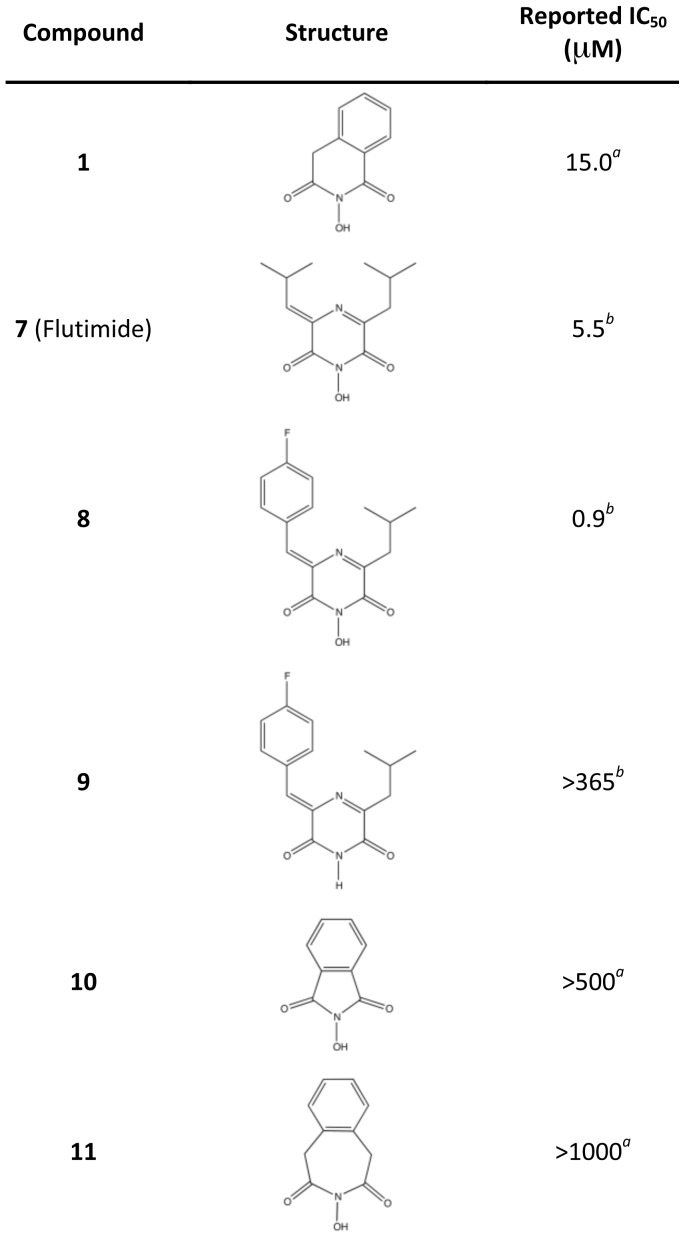
Reported IC_50_ values of Flutimide, Flutimide-related, and *N*-hydroxyimide inhibitors determined in an *in vitro* transcription assay with influenza A polymerase. *^a^*Published results in an influenza virus *in vitro* transcription assay [29]. *^b^*Published results in an influenza virus *in vitro* transcription assay [30].

Figure 5 shows the inhibitory concentration (IC_50_) values of a series of compounds related to **1**, including the natural product inhibitor Flutimide (**7**) [29], [30]. Using the co-crystal structure with **1** (Fig. 4), we analyzed the SAR of this series. We suggest that the increased potency of Flutimide compared with **1** is the result of an interaction between one of the two isobutyl groups and Tyr24, and that this is further enhanced by the larger fluorobenzyl group of **8**, as reflected by the 6-fold increase in potency compared with Flutimide. Docking studies support our hypothesis that compounds **7** and **8** form molecular interactions with Tyr24 (Fig. S5). Finally, the presence and positioning of all three Mn^2+^-binding oxygen atoms is confirmed by the lack of potency observed in compounds **9**–**11**.

**Figure 6:**
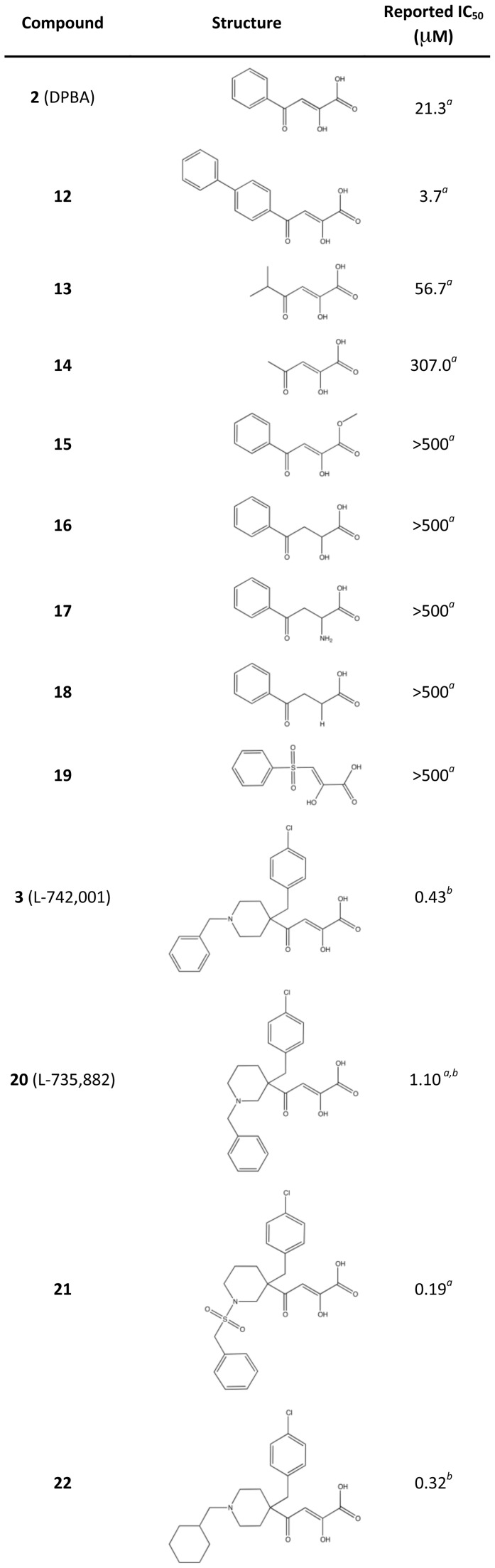
Reported IC_50_ values of 4-substituted 2,4-dioxobutanoic acid inhibitors determined in an *in vitro* transcription assay with influenza A polymerase. *^a^*Published results in an influenza virus *in vitro* transcription assay [15]. *^b^*Published results in an influenza virus *in vitro* transcription assay [26].

Our co-crystal structures with **2** and **3** also provide molecular insights into the SAR of several 4-substituted 2,4-dioxobutanoic acids (Fig. 6) [15], [26]. The addition of an extra phenyl group to **2** as seen in **12** results in a 6-fold gain in potency, and this can be rationalized by additional interactions with Tyr24. Consistent with this, replacement of the phenyl group in **2** with shorter hydrophobic groups in **13** and **14** results in 2.6- and 14-fold reductions in potency, respectively. The importance of the electrostatic interaction between the carboxyl group and Lys134 is confirmed by **15**, in which the replacement of the carboxyl with a methyl ester severely compromises potency. Similar to the effect seen in the Flutimide-related compounds, deletion or repositioning of metal-coordinating oxygen atoms eliminates activity (**16–19**). Compounds **20, 21** and **22** were found to inhibit *in vitro* transcription and endonuclease activity with high potency similar to **3** (Fig. 6), and to exhibit dose-dependent inhibition of viral replication in cell culture [15], [26]. While the additional groups at the 4-position of the dioxobutanoic acid scaffold clearly increase the activity of these compounds, the differences between our structure with compound **3** and the structures in the accompanying article with **20, 21** and **22** [31] make it difficult to characterize their SAR. However, the observed conformational differences do suggest that the potencies of these compounds can be significantly improved now that structural information is available.

**Figure 7:**
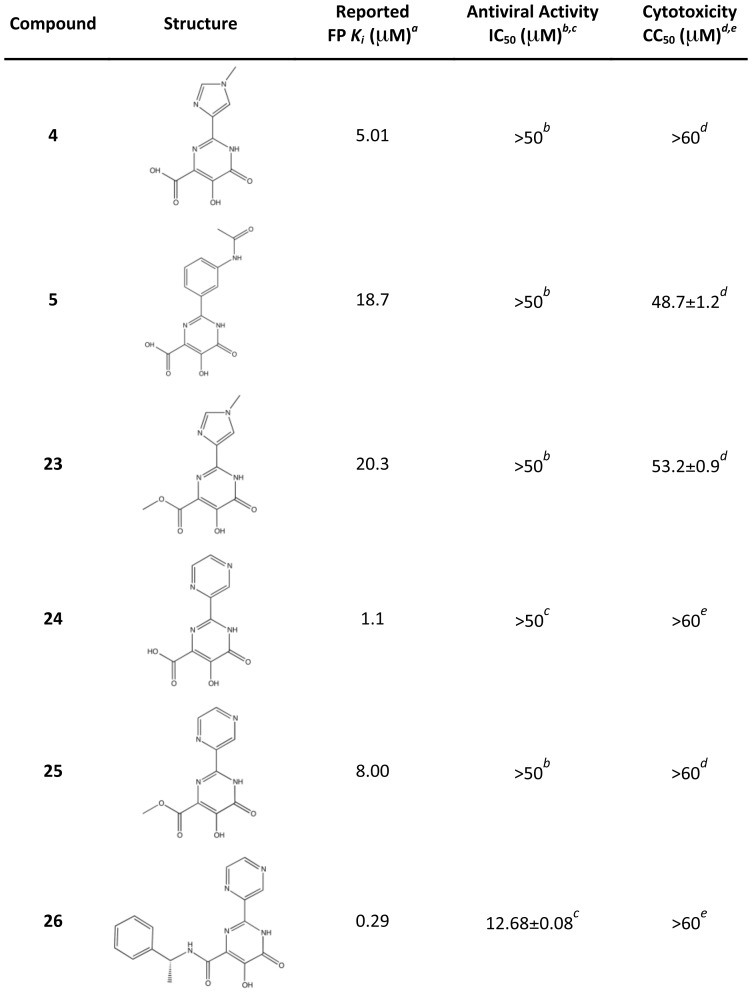
Reported PA_N_ binding activities, antiviral activities, and cytotoxicities of compounds 4 and 5 and related compounds. *^a^*Published results in a competitive binding fluorescence polarization assay with PA_N_ [40]. ^b,c^Antiviral activity as measured by inhibition of viral plaque formation in this study*^b^* or previously*^c^* [40]. *^d,e^*Compound cytotoxicity in MDCK cells after 72 hours as measured in this study*^d^* or previously*^e^* [40].

Finally, we recently used a fluorescence polarization assay to identify several additional PA_N_ inhibitors that are related to **4** and **5** (Fig. 7) [40]. In compounds **23**, **25** and **26**, the carboxylic acid has been replaced with marginal impact on potency as reflected in the *K_i_* values. This is consistent with the co-crystal structures of **4** and **5**, in which the carboxylic acid does not interact with Lys134 and there is available space for the substituent (Fig. 4). The significant gain in potency of **26** may reflect an interaction with Tyr24 as observed in **2** and **3** (Fig. 4). The increase in potency of **26** is also reflected in the increase in antiviral activity of this compound (Figs. 7, S6).

## Discussion

Our studies, and those described by Kowalinski and coworkers in the accompanying article [31], provide the first molecular insights into the mechanism of inhibition of the essential influenza enzyme PA endonuclease, and we have confirmed that it represents an ideal target for drug discovery. Previous mutagenesis studies have shown a direct correlation between PA_N_ endonuclease activities and RdRp transcription activities, suggesting that the isolated PA_N_ domain contains the same structure in the context of the intact RdRp [12], [17], [19]. Our biochemical studies show that inhibitors of RdRp transcription also inhibit PA_N_ endonuclease activity, and this validates the use of the isolated PA_N_ endonuclease domain for drug development.

**Figure 8:**
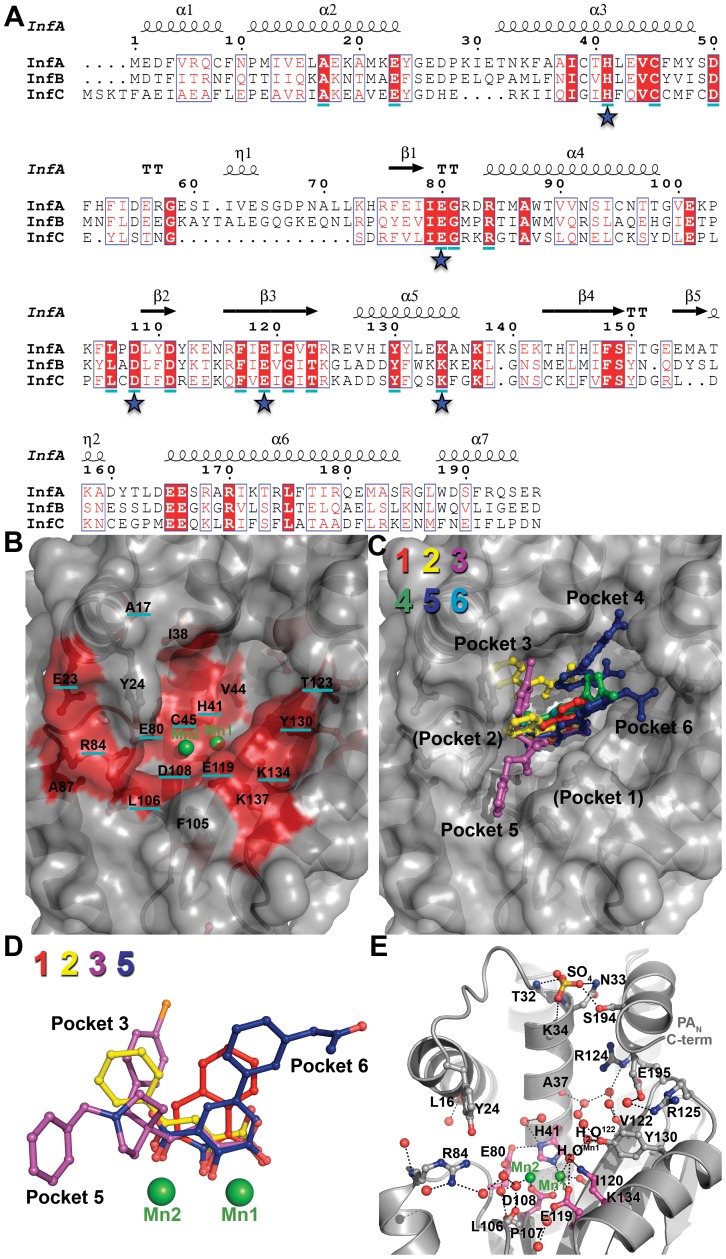
Conserved residues and ordered water molecules in the PA_N_ active site cleft. (**A**) Sequence alignment of PA_N_ from influenza A, B, and C. Consensus sequences were determined from more than 13,000 sequences using the online database www.fludb.org. The secondary structure of PA_N_ from influenza A is shown above the sequence alignment. Residues in a solid red background are identical between the influenza A, B, and C consensus sequences. Residues that are >99.9% conserved in all sequences analyzed are underlined in cyan. Stars indicate key active site residues. (**B**) Surface representation of the PA_N_^ΔLoop^ active site cleft. Manganese ions (Mn1 and Mn2) are shown as green spheres. The highly conserved cleft is colored red. Residues that are identical between influenza A, B, and C consensus sequences are not underlined, and residues that are >99.9% conserved are underlined in cyan. (**C**) Surface representation of PA_N_^ΔLoop^ active site cleft with overlays of compounds **1** (red), **2** (yellow), **3** (purple), **4** (green), **5** (blue), and **6** (cyan). The compounds occupy four (3–6) of the six binding pockets discussed in the text. The orientation and depicted surface are identical to those shown in panel **B**. (**D**) Superposition of bound compounds **1, 2, 3 and 5** (same coloring as panel **C**) after structural alignment of the entire PA_N_^ΔLoop^ domain. For clarity, molecules B of **2** and **5** are not shown. Also not shown for clarity are compounds **4** and **6** because their metal-binding scaffolds overlay perfectly with **5**. Manganese ions (Mn1 and Mn2) are shown as green spheres. (**E**) Ordered water molecules found in at least three of the four molecules in the crystallographic asymmetric unit are shown as red spheres. PA_N_^ΔLoop^ is shown as gray cartoon. Manganese ions (Mn1 and Mn2) are shown as green spheres. Key active site residues are shown in magenta carbon atoms and other residues interacting with the compound or discussed in the text are shown in gray carbon atoms. A sulfate ion is shown as ball-and-stick and colored yellow (sulfur) and red (oxygen). This sulfate is bound at the same location as a predicted phosphate group of nucleic acid in a model of the PA_N_-substrate complex [25]. Black dotted lines represent molecular interactions less than 3.2 Å away.

Our structural studies provide the framework to develop novel inhibitors of the influenza virus PA endonuclease. However, two-metal active sites are ubiquitous in enzymes that process nucleic acids, and it may be challenging to develop drugs that specifically target PA_N_ endonuclease. We therefore analyzed the PA_N_ active site for conserved and unique features for drug discovery by aligning ∼13,000 PA amino acid sequences to identify the consensus sequence for PA_N_ of influenza types A, B, and C (Fig. 8A). Thirty residues are highly conserved and 17 are more than 99.9% identical. Unsurprisingly, most are in the active site pocket and include the metal-binding residues His41, Glu80, Asp108, and Glu119 and the catalytic residue Lys134 (Fig. 8B). The central scaffolds of our characterized inhibitors interact with these residues and are likely to be resistant to mutation but are unlikely to be useful for specificity.

Our studies have shown that interactions with residues further away from the two-metal center substantially increase potency. The same conclusion has been drawn by Kowalinski and coworkers who specifically identified four pockets that can be exploited for inhibitor optimization [31]. Figure 8C maps out how compounds **1**–**6** engage these pockets, and it can be seen that none of the compounds bind pockets 1 and 2, which only appear to become available upon side-chain rotation and inhibitor binding [31]. However, our structures reveal two additional pockets 5 and 6. Compounds **2** and **3** occupy pocket 3 and interact with Tyr24, which is a highly conserved aromatic residue. The biological role of Tyr24 is revealed in the studies of Kowalinski and coworkers which show that it forms a crucial stacking interaction with the base of the mononucleotide [31]. The new pocket 5 is revealed by the binding of the benzylpiperidine group of compound **3**; it comprises conserved residues Arg84, Trp88, Phe105, and Leu106, and is an excellent target for further exploration (Figs. 4C, 8C). The same is true for the new pocket 6 that engages the acetamide group of compound **5** and comprises highly conserved residues Thr123, Tyr130, Lys134 and Lys137 (Figs. 4E, 8C). Mutation of Arg84, Tyr130, or Lys137 to Ala reduces but does not eliminate endonuclease activity, suggesting that inhibitor resistance could develop, although possibly at a cost to virus fitness [12], [19]. Similarly, the interactions between molecule B of compound **5** and pocket 4 residues Lys34 and Arg124 are unlikely to be useful for drug development because these residues are not well conserved. However, π-stacking interactions have been shown to be very productive in terms of increasing potency [35], [41], [42], and Tyr24, His41, F105, Tyr130, and F150 offer potential opportunities. These data reveal the potential for the use of growing and linking strategies to design potent inhibitors.

The entropic contribution to binding can be substantial when ordered water molecules are displaced [43], [44], [45], and the PA_N_ active site offers opportunities in this regard. PA_N_ contains a large, deep active site (over 3000 Å^3^) with several ordered water molecules, 17 of which are found in at least three of the four PA_N_ molecules in the asymmetric unit (Fig. 8E). A large network of water molecules near Val122 becomes displaced by molecule B of compound **5**, and a network of four water molecules between Mn2 and Arg84 is displaced by the benzylpiperidine group of compound **3**, and both can be targeted for inhibitor optimization. Ordered water molecules can also be mimicked by oxygen atoms introduced during inhibitor optimization (see for example [46]). Our studies provide an example of this. One water molecule (H_2_O^Mn1^) that interacts with Mn1, Glu119, and Lys134 becomes displaced by an oxygen atom from compounds **1**–**6** (Figs. 8D, 8E). H_2_O^Mn1^ also forms a hydrogen bond with water molecule H_2_O^122^, which in turn forms hydrogen bonds with Val122 (backbone amide), Tyr130, and another water molecule. Modification of inhibitors that displace H_2_O^122^ but preserve its hydrogen bonds should significantly improve inhibitor binding via gains in both entropy and enthalpy.

Another important consideration in the design of optimal inhibitors is the location and coordination sphere of each Mn^2+^ ion in the PA_N_ active site. Detailed structural analyses on the *Bacillus halodurans* RNase H revealed that the distance between the metal ions changes at different stages of phosphodiester hydrolysis [47], [48]. Consistent with this is the observation that the metals are approximately 2.9 Å apart in PA_N_^ΔLoop^–Apo and move to 3.8–4.0 Å apart when an inhibitor is bound. This mobility seems to occur in Mn2 because Mn1 is in a similar location in both the unbound and inhibitor-bound structures. Our data suggest that the inhibitor-bound form of PA_N_ represents the enzyme-substrate complex stage in which the metals are separated by about 4.0 Å [47], [48]. Thus, computational modeling or docking of inhibitors may best be suited with the inhibitor-bound form of PA_N_ and Mn^2+^ ions.

Furthermore, metal coordination appears to play an important role in compound binding. Specifically, the compound oxygen atoms that coordinate Mn1 in all the complexes described here and in the accompanying article [31] are separated by two atoms (Fig. 8D), and this allows them to ideally contribute to the octahedral geometry completed by the Mn1-coordinating oxygen atoms from H41, D108, E119, and I120.

Finally, our studies support the potential for developing antiviral inhibitors that target the endonuclease activity of other negative strand and cap-snatching segmented RNA viruses, specifically the *Orthomyxoviridae*, *Bunyaviridae*, and *Arenaviridae* families. Recent crystal structures of the endonuclease domains from La Crosse orthobunyavirus L protein and lymphocytic choriomeningitis virus L protein reveal clear structural homology to the influenza A virus PA_N_ endonuclease domain with dependence on manganese ions for activity [32], [49] (Fig. S7). However, low sequence homology and structural variation between virus family endonucleases suggest opportunities for developing virus family-specific inhibitors.

## Methods

### Chemical Synthesis of Inhibitors

The activity, but not synthesis, of compound **1** (an *N*-hydroxyimide) was described previously [29]. We produced compound **1** using synthetic conditions described by Birch et al. [50]. Briefly, hydroxylamine HCl (0.9 M) was added to anhydride (1.0 M) in pyridine in a microwavable vessel. The reaction was incubated under a nitrogen atmosphere at 120°C for 60 min under high absorption in a Biotage initiator 60 microwave. Methyl *tert*-butyl ether was used to precipitate the hydroxylsuccinate product that was isolated via filtration. Compound **1** was further re-crystallized with methanol:chloroform. Compounds **2** (2,4-dioxo-4-phenylbutanoic acid, or DPBA) and **3** (L-742,001) were prepared with a slight modification to published methods [15]. Instead of producing a methyl ester intermediate, a *tert*-butyl ester intermediate was produced and then converted to the acid form with trifluoroacetic acid. Compound **4** (5-hydroxy-2-(1-methyl-1H-imidazol-4-yl)-6-oxo-1,6-dihydropyrimidine-4-carboxylic acid) and compound **5** (2-(3-acetamidophenyl)-5-hydroxy-6-oxo-1,6-dihydropyrimidine-4-carboxylic acid) were synthesized in a similar manner as related compounds described previously [37], [51]. Compound **6** (dihydroxybenzoic acid) was purchased from Sigma-Aldrich and used without further purification.

Compound purities were determined by ultra-high-pressure liquid chromatography on a BEH C18 column with a gradient elution of solvent A (0.1% formic acid in water) to solvent B (0.1% formic acid in acetonitrile) using an evaporative light scattering detector (ELSD) and an ultraviolet (UV, 210 to 400 nm) detector. Purities are: compound **1** (ELSD: >99%, UV: 97%), compound **2** (ELSD: 92%, UV: 85%), compound **3** (ELSD: >99%, UV: 98%), compound **4** (ELSD: >99%, UV: 81%), compound **5** (ELSD: >99%, UV: 97%), and compound **6** (ELSD: >99%, UV: 92%). Nuclear magnetic resonance (NMR) spectra measured on a Brooker-400 (400 MHz) spectrometer showed that all compounds are consistent with their assigned structures. NMR experimental results have previously been published [40].

The tautomeric form of compound **2** shown in Figure 2 was confirmed by solving the high resolution (0.84 Å) x-ray crystal structure of the compound alone.

### Cloning

PA_N_ (residues 1–209) or PA_N_^ΔLoop^ (residues 1–50 and 73–196 with a 3-residue linker Gly-Gly-Ser between residues 50 and 73) from H5N1 influenza virus A/Vietnam/1203/2004 (Accession #AY818132) was cloned between the NcoI and NotI sites in the pET52b plasmid in-frame with a C-terminal thrombin cleavage site followed by a 10-histidine purification tag.

### PA_N_ and PA_N_^ΔLoop^ Protein Production

PA_N_ and PA_N_^ΔLoop^ were expressed and purified with modifications to previously published methods [16]. The recombinant proteins were overexpressed in *E. coli* strain BL21 (DE3), and the proteins were purified from soluble lysates by HisTrap affinity chromatography. The 10-histidine purification tags were removed by digestion with biotinylated thrombin, which was later removed by incubation with streptavidin-agarose beads. Undigested protein was removed with cobalt-NTA beads. PA_N_ and PA_N_^ΔLoop^ were then purified by size-exclusion chromatography on a Superdex 75 column in 10 mM Tris pH 8.0, 100 mM NaCl, and 1 mM DTT. Proteins were concentrated to 5–10 mg/ml.

### *In Vitro* Endonuclease Activity Assay

*In vitro* endonuclease activity assays were done with modifications to previously published methods [16]. Single-stranded DNA plasmid M13mp18 (50 ng/µl) was incubated in digestion buffer (10 mM Tris pH 8.0, 100 mM NaCl, 10 mM β-mercaptoethanol, and 2.5 mM MnCl_2_) in the presence of 3, 10, or 30 µM PA_N_ or PA_N_^ΔLoop^ for 2 h at 37°C. The reaction was stopped by adding 50 mM EDTA. For studies with inhibitors, 10 mM inhibitor in DMSO was diluted 3-fold in series with DMSO and then used at a 10% concentration in enzymatic reactions containing 15 µM PA_N_. Reaction products were resolved on a 1.0% agarose gel stained with ethidium bromide.

### Crystal Structure Determination

PA_N_^ΔLoop^ protein crystals were grown by the hanging-drop vapor diffusion method at 18°C in a well solution of 1.50 M ammonium sulfate, 2% PEG1500, 0.1 M Tris pH 8.0, and 1 mM MnCl_2_. Crystals grew after 3–4 days. Crystals were transferred into a soak solution (1.65 M ammonium sulfate, 2% PEG1500, 0.1 M Tris pH 8.0, 5 mM MnCl_2_, and 10 mM MnCl_2_) containing ∼20 mM inhibitor and incubated overnight at 18°C. Crystals were quickly transferred into a cryo-protection solution (0.4 M ammonium sulfate, 2% PEG1500, 0.1 M Tris pH 8.0, 5 mM MnCl_2_, 10 mM MnCl_2_, and 25% PEG400) containing 10 mM inhibitor before flash freezing in liquid nitrogen. In the case of PA_N_^ΔLoop^-Apo, crystals were mock-soaked in soak solution without inhibitor and cryo-protected without inhibitor.

Diffraction data were collected at cryogenic temperature at X-ray wavelength 1.00 Å from the Southeastern Regional Collaborative Access Team's 22-ID and 22-BM beamlines at the Advanced Photon Source (Argonne National Laboratory, Chicago, IL). Data processing and reduction were completed with HKL-2000 software [52].

The PA_N_^ΔLoop^-Apo structure was determined by molecular replacement using the program Phaser [53]. A solution was obtained by using a model of the avian PA_N_ crystal structure (PDB code 3EBJ, residues 1–50 and 73–196) [17]. The model was corrected to encode PA residues from A/Vietnam/1203/2004, and residues 80, 108, and 119 were mutated to alanine to remove model bias from these metal-coordinating active-site residues. Simulated annealing was then done using Phenix [54]. Residues 80, 108, and 199 were corrected and model building was performed using Coot [55] followed by restrained refinement using the CCP4 software suite's REFMAC5 [56]. Refinement was monitored by following the R_free_ value calculated for a random subset (5%) of reflections omitted from refinement. For the PA_N_^ΔLoop^-inhibitor structures, simulated annealing was done with PA_N_^ΔLoop^-Apo without Mn^+2^ ions and with residues 80, 108, and 119 mutated to alanine to remove model bias.

### Isothermal Titration Calorimetry Assays

Purified PA_N_ protein was dialyzed against 25 mM HEPES pH 8.0, 100 mM NaCl, and 1 mM MnCl_2_. ITC titrations were performed with an Auto-iTC200 Isothermal Titration Calorimeter (MicroCal) at 25°C. Nineteen injections of 2 µl each of 2 mM compound **2** were titrated into 100 µM protein solution. 5% DMSO was added to the ITC buffer for the titration experiment. Data were analyzed using MicroCal Origin 7.0 software using a One-Site binding model and Sequential Binding Sites model with two sites. The experiments were performed independently twice and showed very similar results.

### Docking of Compounds

Docking of compounds **3**, **7** (Flutimide), and **8** into PA_N_^ΔLoop^ active site was performed by Glide module in Schrodinger software. For compound **3**, the docking model was generated from the crystal structure of the PA_N_^ΔLoop^–compound **2** complex, with the 2,4-dioxobutanoic acid group defined as the reference core structure for guiding the corresponding functional group in compound **3** into the correct orientation (tolerance set to 0.8 Å RMSD). For compounds **7** and **8**, the docking model was generated from the crystal structure of the PA_N_^ΔLoop^–compound **1** complex, with the *N*-hydroxyimide group defined as the reference core structure for guiding the corresponding functional group in compounds **7** and **8** into the correct orientation (tolerance set to 0.8 Å RMSD). Two Mn^2+^ ions in the active site were kept as part of the protein. The binding pocket is defined as residues within 20 Å radius of the reference core structure. All water molecules were deleted from the protein structure before docking. The compound geometries were built and optimized by SYBYL program. The standard precision of Glidescore scoring functions was used to rank binding poses.

### Antiviral Activity Assays

Antiviral activity assays were carried out exactly as done previously [40]. Briefly, avian H1N1 influenza A virus (A/PuertoRico/8/34) grown in embryonated eggs was used for infection [50–100 PFU of PR8 virus per well (MOI = 0.0001)] in Madin-Darby canine kidney (MDCK) cells (3×10^5^ cells/well). After 1 h, each well was overlaid with medium containing agarose and compound (at least 10 concentrations of each compound). After 72 h, plaques were visualized with crystal violet and counted. The concentration of compound required for 50% inhibition of plaque formation (IC_50_) was determined for triplicate measurements by nonlinear least-squares analysis using GraphPad Prism 4.03.

### Compound Cytotoxicity Assays

Compound cytotoxicity assays were carried out exactly as done previously [40]. Briefly MDCK cells (3×10^5^ cells/mL, 20 µL per well) were incubated with compound at 2-fold serial dilutions from 60 µM. The negative control was 0.6% DMSO and the positive control was 60 µM staurosporine. After 72 h, 20 µL CellTiter-Glo reagent was added and luminescence was measured. The concentration of compound required to decrease cell viability by 50% (CC_50_), was determined for triplicate measurements by nonlinear least-squares analysis using GraphPad Prism 4.03.

### Accession Numbers

The atomic coordinates and structure factors have been deposited in the Protein Data Bank, www.pdb.org, under accession numbers 4E5E, 4E5F, 4E5G, 4E5H, 4E5I, 4E5J, and 4E5L.

## Supporting Information

Figure S1 PA_N_^ΔLoop^ crystal packing and active site manganese ions. (**A**) Four PA_N_^ΔLoop^ molecules in the crystallographic asymmetric unit. The Gly-Gly-Ser linker that replaces a 22 amino acid loop is shown as magenta spheres. Manganese ions in the active sites are shown as green spheres. (**B**) Simulated-annealing Fo-Fc omit map (brown) contoured at 3.0 σ around the manganese ions in the PA_N_^ΔLoop^ active site from crystals soaked in the absence of magnesium ions.
(TIF)

Figure S2 Electron densities of compounds **1**–**6** (**A**–**F**, respectively). Each panel shows the final 2Fo-Fc electron density map (blue) and the simulated-annealing Fo-Fc omit map (brown) contoured at 1.0 σ and 3.0 σ, respectively. PA_N_^ΔLoop^ is shown as cartoon and colored gray. Compounds are shown as ball-and-stick models and are colored yellow (carbon), blue (nitrogen), red (oxygen), and orange (chlorine). Manganese ions (Mn1 and Mn2) are shown as green spheres.
(TIF)

Figure S3 Induced-fit binding by compounds **2** and **3**. (**A**) Comparison of PA_N_^ΔLoop^-Apo (gray) and PA_N_^ΔLoop^-compound **2** (purple) structures reveals the movement of Tyr24 on helix-α3. Two molecules of compound **2** (yellow labels A and B) are shown as ball-and-stick models and are colored yellow (carbon), blue (nitrogen), and red (oxygen). Manganese ions (Mn1 and Mn2) are shown as green spheres. The gray arrow shows the movement of helix-α3 residue Tyr24. (**B**) Comparison of PA_N_^ΔLoop^-Apo (gray) and PA_N_^ΔLoop^-compound **3** (purple) structures, displayed as in panel **A**. (**C**) PA_N_^ΔLoop^-Apo active site colored by B-factor from blue (B-factor ∼20) to white to red (B-factor ∼50).
(TIF)

Figure S4 Isothermal titration calorimetry (ITC) binding of PA_N_ and compound **2**. (**A**) One-site model. (**B**) Sequential binding site model with two sites. In the lower panels, the solid squares represent experimental data, and the continuous lines correspond to the model fits. Note that binding by compound **2** is endothermic and is entropically favorable, possibly by displacement of water molecules shown in Figure 8E.
(TIF)

Figure S5 Docking models of **3**, **7** (Flutimide), and **8** in the PA_N_ active site. (**A–B**) Comparison of the crystal structure with compound **3** (**A**) and the docked model with compound **3** (**B**). (C–F) Comparison of the crystal structure with compound **1** (**C**) and the docked model with compound **7** (Flutimide) (**D**) and compound **8** (**E–F**). Panels (**E**) and (**F**) represent two docked orientations of compound **8**. In all panels, PA_N_^ΔLoop^ is shown as cartoon and colored gray. Manganese ions (Mn1 and Mn2) are shown as green spheres. Tyr24 that is predicted to interact with compounds **7** and **8** is shown as cyan. Compounds are shown as ball-and-stick models and are colored blue (nitrogen), red (oxygen), light orange (chlorine), and violet (fluorine), with yellow and orange carbons, respectively, in the crystal structures and the docked structures. Docking scores for compounds **3**, **7** and **8** are −9.3 kcal/mol, −4.5 kcal/mol, and −5.2 kcal/mol, respectively. Docking scores for compound **8** are the same for the two orientations observed in panels (**E**) and (**F**).
(TIF)

Figure S6 Antiviral activities of compounds listed in Figure 7. Antiviral activity was measured by inhibition of viral plaque formation in MDCK cells after 72 hours. IC_50_ values are reported in Figure 7.
(TIF)

Figure S7 Endonuclease domains from other cap-snatching RNA viruses. Endonuclease domain structures from the influenza A virus PA protein (Orthomyxovirus), La Crosse orthobunyavirus L protein (Bunyavirus), and lymphocytic choriomeningitis virus L protein (Arenavirus). Structures are shown as cartoon and colored blue-to-red rainbow from N- to C-termini. Key active site residues are colored magenta and are shown as ball-and-stick. The coordinates for the bunyavirus and arenavirus structures are from PDB entries 2XI5 and 3JSB, respectively.
(TIF)
