# Supplementary figures and images for: Structural and Biochemical Basis for Development of Influenza Virus Inhibitors Targeting the PA Endonuclease
Source: PLoS Pathog. 2012 Aug 2;8(8):e1002830. doi: 10.1371/journal.ppat.1002830 (PMC3410894; doi:10.1371/journal.ppat.1002830)

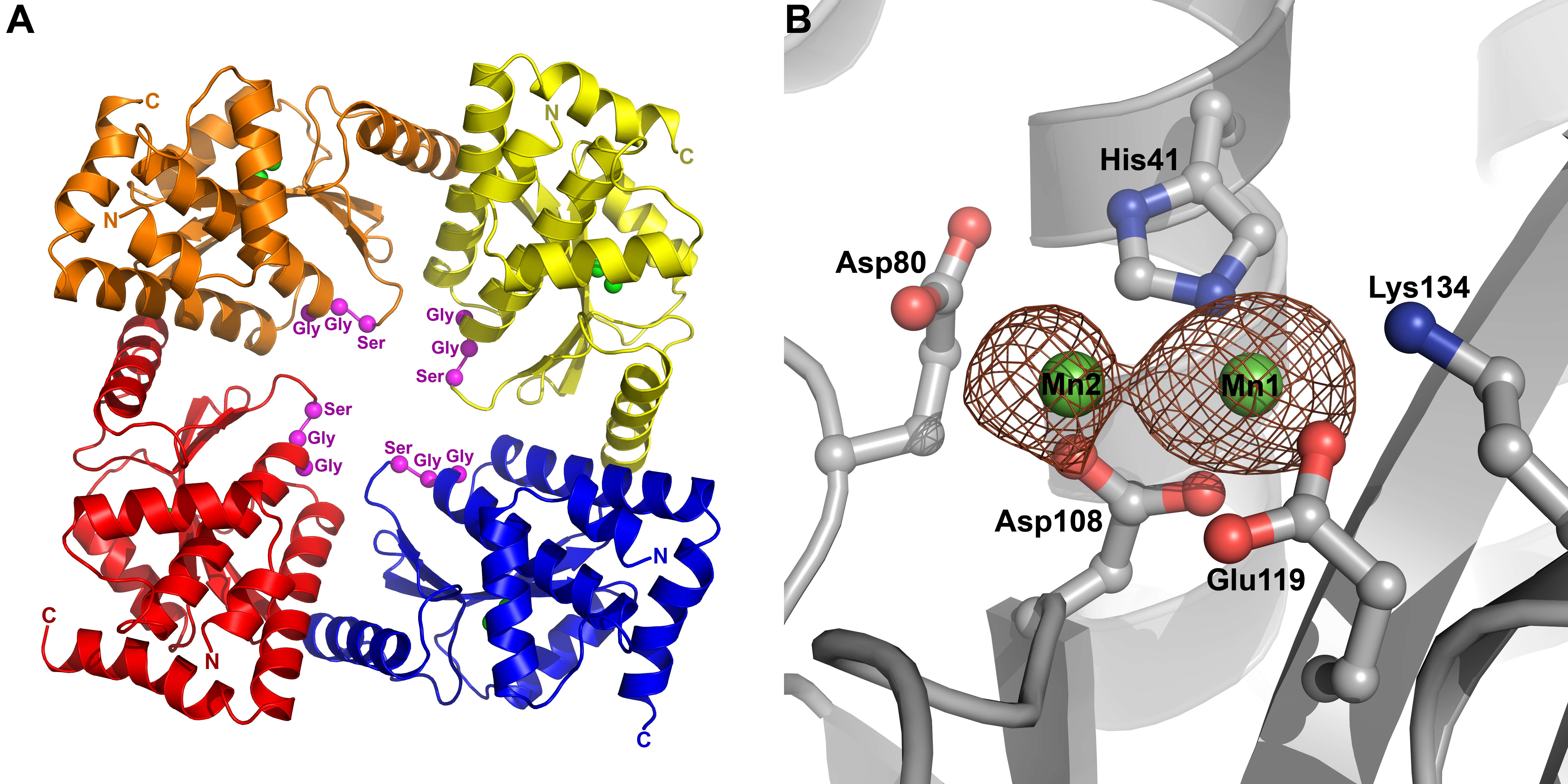

Supplement: Figure S1 — PAN ΔLoop crystal packing and active site manganese ions. (A) Four PAN ΔLoop molecules in the crystallographic asymmetric unit. The Gly-Gly-Ser linker that replaces a 22 amino acid loop is shown as magenta spheres. Manganese ions in the active sites are shown as green spheres. (B) Simulated-annealing Fo-Fc omit map (brown) contoured at 3.0 σ around the manganese ions in the PAN ΔLoop active site from crystals soaked in the absence of magnesium ions. (TIF) [file ppat.1002830.s001.tif]

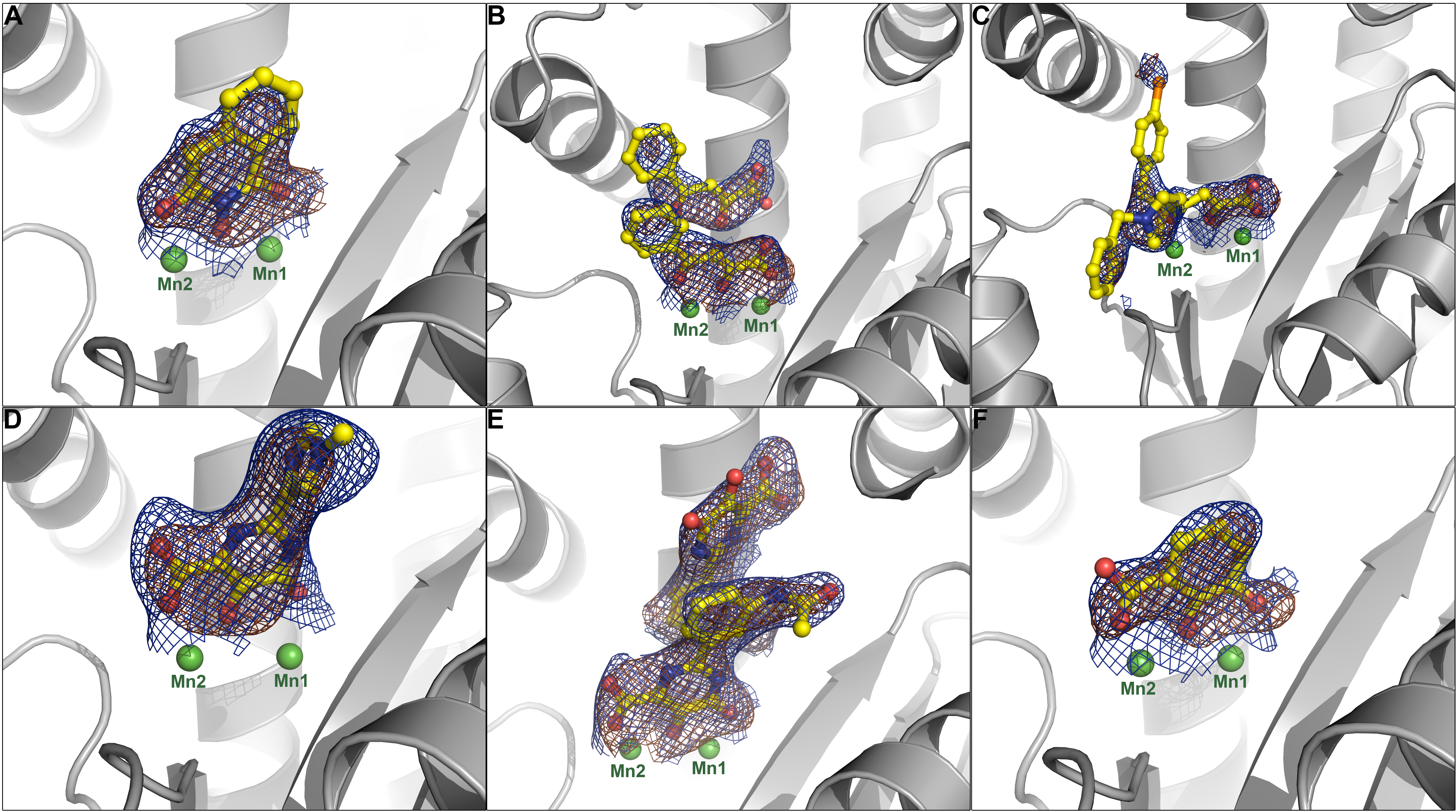

Supplement: Figure S2 — Electron densities of compounds 1–6 (A–F, respectively). Each panel shows the final 2Fo-Fc electron density map (blue) and the simulated-annealing Fo-Fc omit map (brown) contoured at 1.0 σ and 3.0 σ, respectively. PAN ΔLoop is shown as cartoon and colored gray. Compounds are shown as ball-and-stick models and are colored yellow (carbon), blue (nitrogen), red (oxygen), and orange (chlorine). Manganese ions (Mn1 and Mn2) are shown as green spheres. (TIF) [file ppat.1002830.s002.tif]

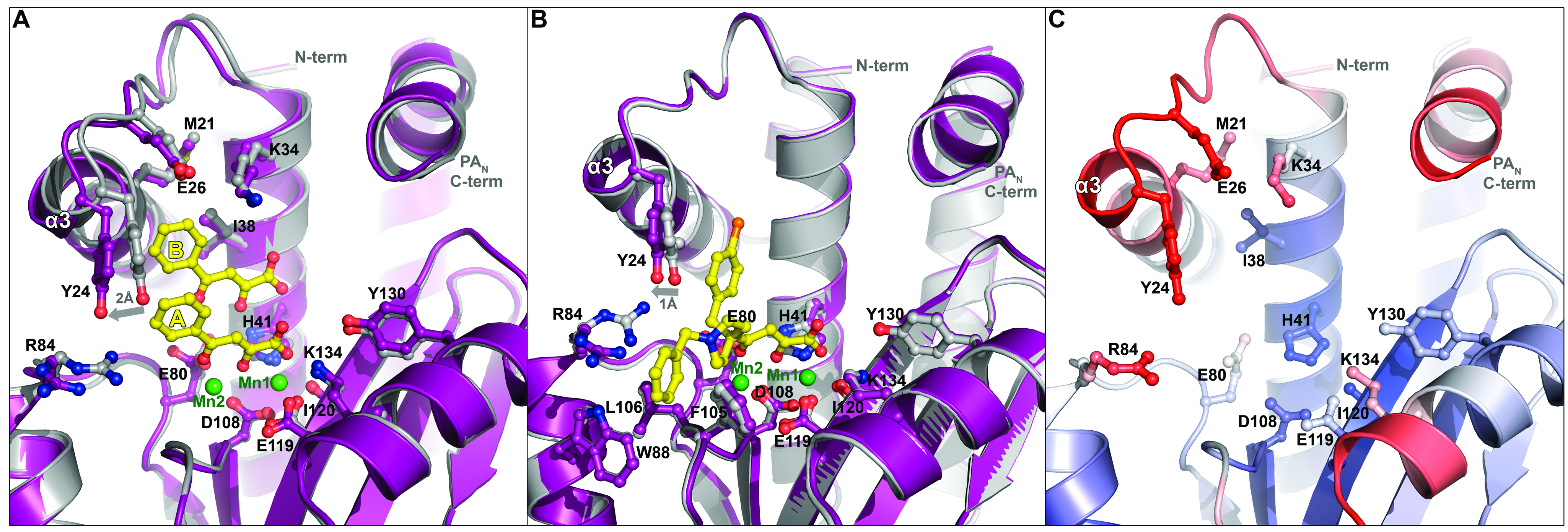

Supplement: Figure S3 — Induced-fit binding by compounds 2 and 3. (A) Comparison of PAN ΔLoop-Apo (gray) and PAN ΔLoop-compound 2 (purple) structures reveals the movement of Tyr24 on helix-α3. Two molecules of compound 2 (yellow labels A and B) are shown as ball-and-stick models and are colored yellow (carbon), blue (nitrogen), and red (oxygen). Manganese ions (Mn1 and Mn2) are shown as green spheres. The gray arrow shows the movement of helix-α3 residue Tyr24. (B) Comparison of PAN ΔLoop-Apo (gray) and PAN ΔLoop-compound 3 (purple) structures, displayed as in panel A. (C) PAN ΔLoop-Apo active site colored by B-factor from blue (B-factor ∼20) to white to red (B-factor ∼50). (TIF) [file ppat.1002830.s003.tif]

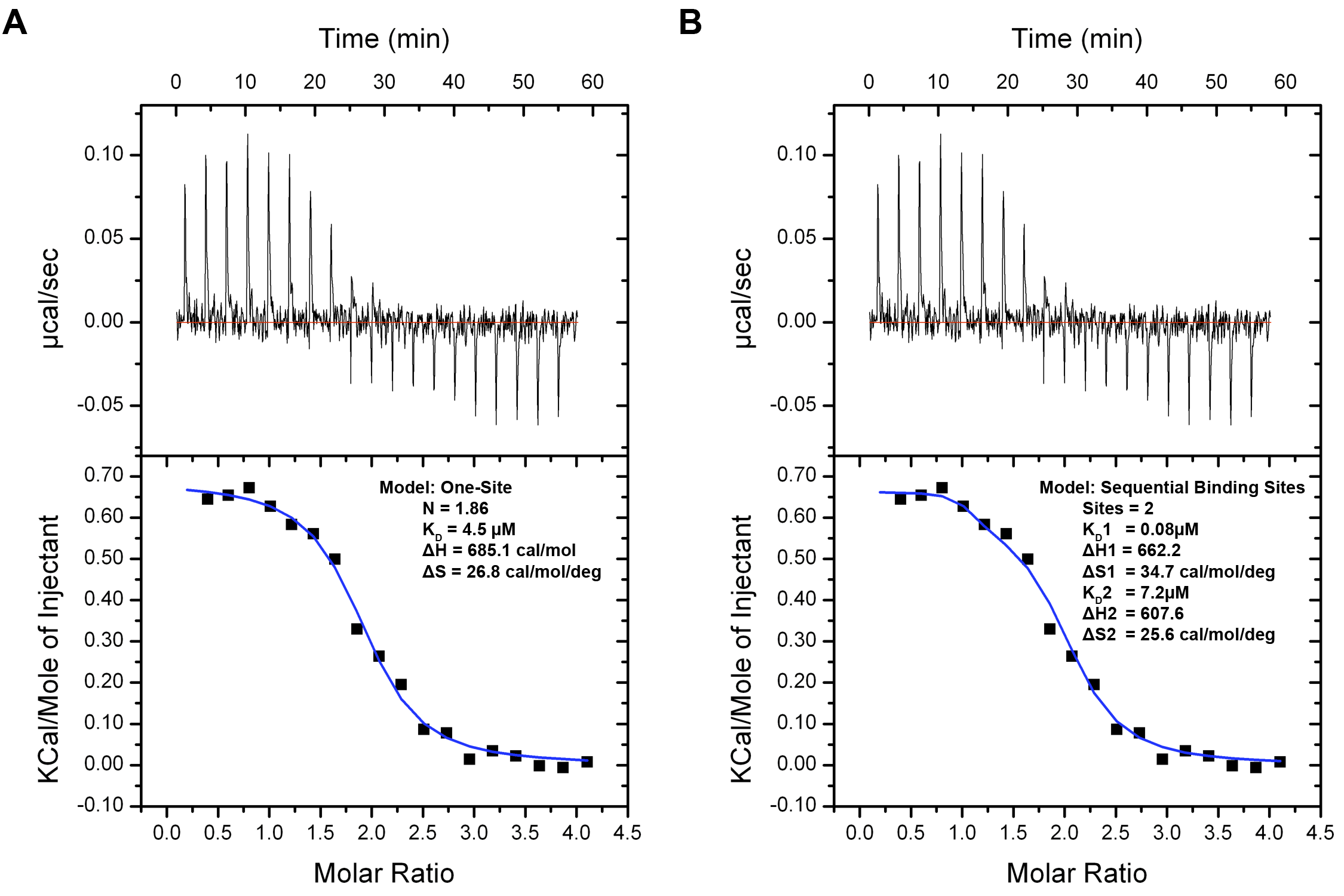

Supplement: Figure S4 — Isothermal titration calorimetry (ITC) binding of PAN and compound 2. (A) One-site model. (B) Sequential binding site model with two sites. In the lower panels, the solid squares represent experimental data, and the continuous lines correspond to the model fits. Note that binding by compound 2 is endothermic and is entropically favorable, possibly by displacement of water molecules shown in Figure 8E. (TIF) [file ppat.1002830.s004.tif]

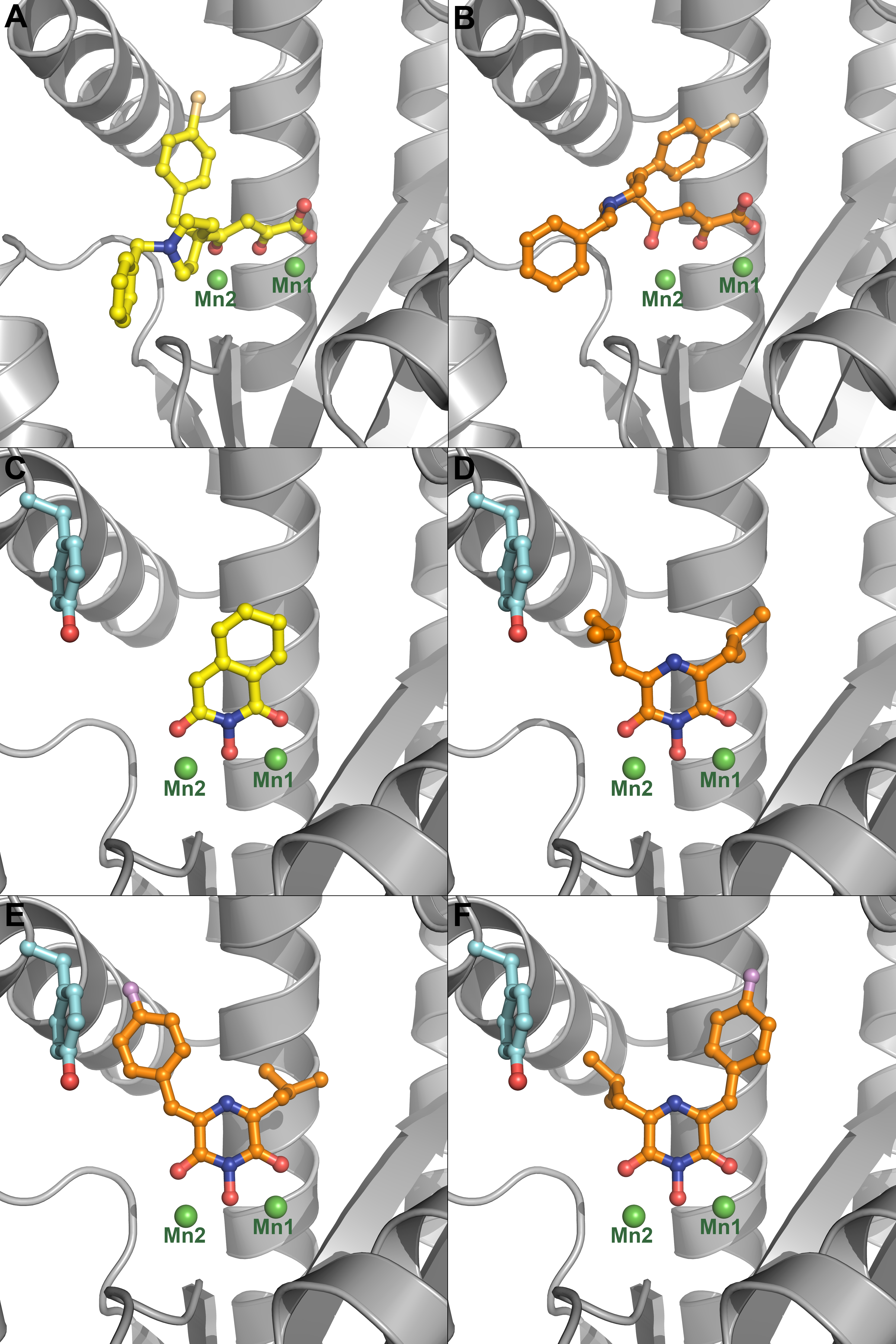

Supplement: Figure S5 — Docking models of 3, 7 (Flutimide), and 8 in the PAN active site. (A–B) Comparison of the crystal structure with compound 3 (A) and the docked model with compound 3 (B). (C–F) Comparison of the crystal structure with compound 1 (C) and the docked model with compound 7 (Flutimide) (D) and compound 8 (E–F). Panels (E) and (F) represent two docked orientations of compound 8. In all panels, PAN ΔLoop is shown as cartoon and colored gray. Manganese ions (Mn1 and Mn2) are shown as green spheres. Tyr24 that is predicted to interact with compounds 7 and 8 is shown as cyan. Compounds are shown as ball-and-stick models and are colored blue (nitrogen), red (oxygen), light orange (chlorine), and violet (fluorine), with yellow and orange carbons, respectively, in the crystal structures and the docked structures. Docking scores for compounds 3, 7 and 8 are −9.3 kcal/mol, −4.5 kcal/mol, and −5.2 kcal/mol, respectively. Docking scores for compound 8 are the same for the two orientations observed in panels (E) and (F). (TIF) [file ppat.1002830.s005.tif]

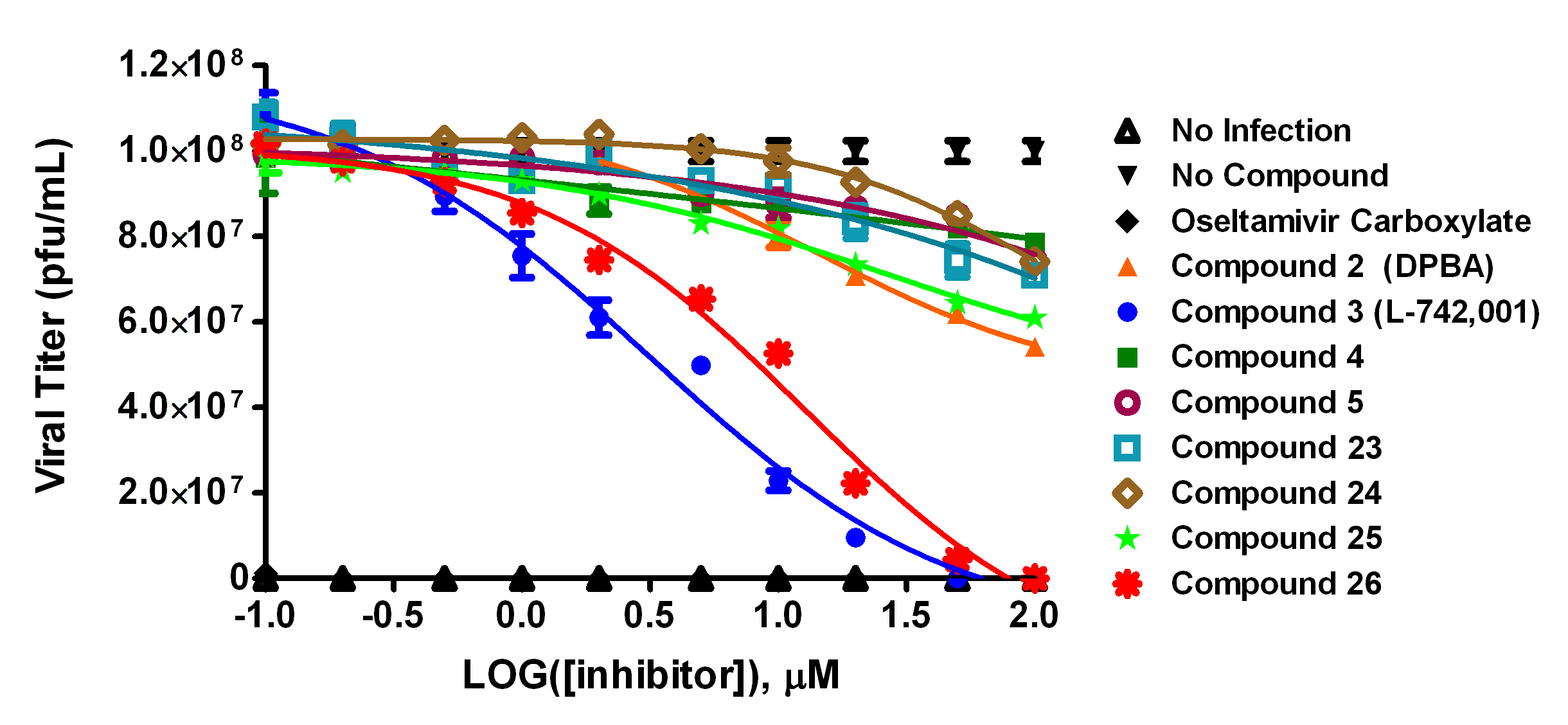

Supplement: Figure S6 — Antiviral activities of compounds listed in Figure 7. Antiviral activity was measured by inhibition of viral plaque formation in MDCK cells after 72 hours. IC50 values are reported in Figure 7. (TIF) [file ppat.1002830.s006.tif]

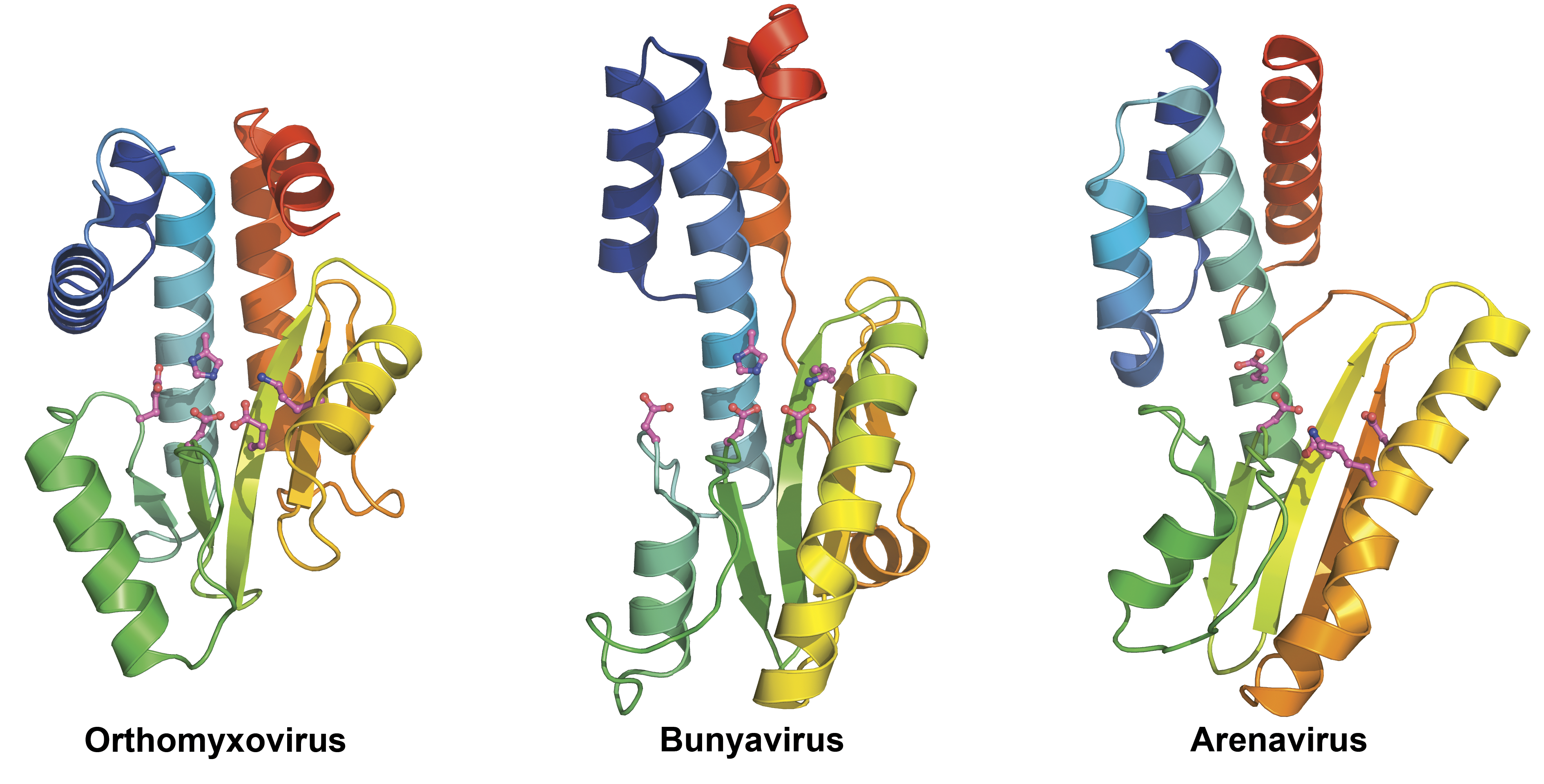

Supplement: Figure S7 — Endonuclease domains from other cap-snatching RNA viruses. Endonuclease domain structures from the influenza A virus PA protein (Orthomyxovirus), La Crosse orthobunyavirus L protein (Bunyavirus), and lymphocytic choriomeningitis virus L protein (Arenavirus). Structures are shown as cartoon and colored blue-to-red rainbow from N- to C-termini. Key active site residues are colored magenta and are shown as ball-and-stick. The coordinates for the bunyavirus and arenavirus structures are from PDB entries 2XI5 and 3JSB, respectively. (TIF) [file ppat.1002830.s007.tif]
